# Improved tolerance to drought stress after anthesis due to priming before anthesis in wheat (*Triticum aestivum* L.) var. Vinjett

**DOI:** 10.1093/jxb/eru362

**Published:** 2014-09-09

**Authors:** Xiao Wang, Marija Vignjevic, Dong Jiang, Susanne Jacobsen, Bernd Wollenweber

**Affiliations:** ^1^Key Laboratory of Crop Physiology and Ecology in Southern China, Ministry of Agriculture/Hi-Tech Key Laboratory of Information Agriculture of Jiangsu Province, Nanjing Agricultural University, PR China; ^2^Aarhus University, Faculty of Science and Technology, Institute of Agroecology, Research Centre Flakkebjerg, DK-4200 Slagelse, Denmark; ^3^Enzyme and Protein Chemistry, Department of Systems Biology, Technical University of Denmark, Building 224, DK-2800 Kgs. Lyngby, Denmark

**Keywords:** Drought tolerance, leaf proteome, photosynthesis, priming, wheat.

## Abstract

Drought stress occurring during the reproductive growth stage leads to considerable reductions in crop production and has become an important limiting factor for food security globally. In order to explore the possible role of drought priming (pre-exposure of the plants to mild drought stress) on the alleviation of a severe drought stress event later in development, wheat plants were subjected to single or double mild drought episodes (soil relative water content around 35–40%) before anthesis and/or to a severe drought stress event (soil relative water content around 20–25%) 15 d after anthesis. Here, single or double drought priming before anthesis resulted in higher grain yield than in non-primed plants under drought stress during grain filling. The photosynthesis rate and ascorbate peroxidase activity were higher while malondialdehyde content was lower in primed plants than in the non-primed plants under drought stress during grain filling. Proteins in flag leaves differently expressed by the priming and drought stress were mainly related to photosynthesis, stress defence, metabolism, molecular chaperone, and cell structure. Furthermore, the protein abundance of ribulose-1,5-bisphosphate carboxylase/oxygenase (Rubisco) small subunit, Rubisco activase and ascorbate peroxidase were upregulated in primed plants compared with non-primed plants under drought stress during grain filling. In conclusion, the altered protein expression and upregulated activities of photosynthesis and ascorbate peroxidase in primed plants may indicate their potential roles in alleviating a later-occurring drought stress episode, thereby contributing to higher wheat grain yield under drought stress during grain filling.

## Summary

Physiological and proteome insights were determined into the alleviating effects of pre-anthesis drought priming on drought stress during post-anthesis, which will be of importance for future drought-tolerance studies in cereals.

## Introduction

It is known that the frequency and duration of extreme climate episodes are increasing (Ciais *et al.*, 2005). Drought events are limiting crop production, and particularly those occurring during the reproductive growth stage can lead to significant reductions in yield and quality (Barnabas *et al.*, 2008; Farooq *et al.*, 2009).

The mechanisms of plant drought tolerance are complex and involve diverse and multiple physiological and molecular mechanisms (Shinozaki and Yamaguchi-Shinozaki, 2007; Farooq *et al.*, 2009). It has been reported that the downregulation of photosynthesis due to mild drought stress is mainly the result of a reduction in stomatal conductance, while the photosynthetic apparatus is not significantly affected (Cornic and Fresneau, 2002; Harb *et al.*, 2010). As a consequence of severe drought stress events, both stomatal and non-stomatal limitations lead to a decline in photosynthesis (Bota *et al.*, 2004; Flexas *et al.*, 2008), For example, electron transport from photosystem II (PSII) to PSI and enzymes of carbon metabolism [e.g. ribulose-1,5-bisphosphate carboxylase/oxygenase (Rubisco) and enzymes related to ribulose-1,5-bisphosphate synthesis] accounted for the lower photosynthesis rate under these conditions (Medrano *et al.*, 2002; Tezara *et al.*, 1999).

The electron transport chain in the chloroplastic thylakoid membrane is the major source of reactive oxygen species (ROS) under stress conditions (Apel and Hirt, 2004). Damage to the PSII oxygen-evolving complex (Canaani *et al.*, 1986) and to the reaction centre (He *et al.*, 1995; Reddy *et al.*, 2004) may lead to the imbalance of electron generation and utilization, resulting in the generation of ROS (Reddy *et al.*, 2004), and consequently cause lipid peroxidation of cell membranes (Murata *et al.*, 2007). Antioxidant enzymes, such as superoxide dismutase, glutathione reductase and ascorbate peroxidase, play important roles in scavenging excess ROS, which are generated by abiotic stress (Jiang and Zhang, 2002; Hernández *et al.*, 2012).

It has been shown that priming—the pre-exposure of plants to an eliciting factor—enables plants to become more tolerant to later-occurring biotic or abiotic stress events (Bruce *et al.*, 2007). To date, many studies have focused on biotic-induced priming and the mechanisms, which include, among others, the accumulation of mitogen-activated protein kinases (MPKs), epigenetic changes, and regulation of primary metabolism (Uwe *et al.*, 2006; Beckers and Conrath, 2007; Bruce *et al.*, 2007; Conrath, 2011). Attention has also been given to chemical-induced priming, such as by nitric oxide (Tanou *et al.*, 2009; Molassiotis *et al.*, 2010), β-aminobutyric acid (Tsai *et al.*, 2011), hydrogen sulfide (Christou *et al.*, 2013; Shi *et al.*, 2013), and acclimation mainly through regulation of defence-related genes/proteins as well as induction of antioxidant mechanisms (Jakab *et al.*, 2005; Filippou *et al.*, 2012; Tanou *et al.*, 2012).

However, fewer studies investigated abiotic stress-induced priming. It was found in *Arabidopsis* that multiple pre-exposures to mild drought stress episodes increased the flexibility of the plant to cope with a recurring drought stress event, and that a stalled RNA polymerase II was involved in the transcriptional drought memory (Ding *et al.*, 2012). However, the duration between priming and the reoccurring stress was very short (several hours or days) (Bruce *et al.*, 2007; Walter *et al.*, 2011). Whether plants are able to conserve the ‘memory’ of a previous stress episode to a subsequent stress event later in development, as well as the underlying mechanisms, is far from clear. Our previous studies have shown that priming with high temperatures (Wang *et al.*, 2011, 2012) or waterlogging (Li C *et al*., 2011) before anthesis could alleviate the negative effects of the same stress occurring after anthesis, as exemplified by improved grain yields in primed plants compared with non-primed plants in wheat. Walter *et al.* (2011) found that plants experiencing an early drought episode showed a higher percentage of biomass and improved photo-protection than non-primed plants under a second drought event in *Arrhenatherum elatius*. However, Zavalloni *et al.* (2008) found that elevated temperature and mild drought applied early in development did not enhance tolerance to a later drought stress event in several grass species.

Proteomics is an important tool both for understanding the mechanisms of plants in response to abiotic stress (Chen and Harmon, 2006; Caruso *et al.*, 2009; Kottapalli *et al.*, 2009; Kamal *et al.*, 2013) and for gaining insight into possible priming mechanisms (Tanou *et al.*, 2012). Thus, it has been shown that salicylic acid induced drought tolerance in wheat seedlings through regulation of the proteins related to signal transduction, stress defence, photosynthesis and metabolism (Kang *et al.*, 2012). However, to the best of our knowledge, proteome analysis has not been applied for revealing the mechanisms of drought priming in response to a later-occurring drought stress event.

In the present study, we first subjected wheat plants to single and/or multiple mild drought priming events before anthesis, and then to a severe drought stress event during grain filling. Our hypothesis was that: (i) drought priming before anthesis would affect the synthesis and/or activities of enzymes related to photosynthesis and stress defence as mechanisms to enhance tolerance to drought stress occurring during the grain-filling stage; and (ii) there is no difference between drought priming once or twice on the alleviating effect of drought stress during grain filling.

## Materials and methods

### Experimental design

A pot experiment was performed outdoors under field conditions at the Research Centre Flakkebjerg, University of Aarhus, Denmark, in 2011. Each pot (height 18cm and diameter 23cm; 90 pots in total) was filled with 800g of a mixture of soil and peat (v/v, 1:3). Six grains of commercial spring wheat (*Triticum aestivum* L. cv. Vinjett) were sown in each pot and thinned to three plants at the three-leaf stage (growth stage 13, according to Lancashire *et al.*, 1991).

**Fig. 1.**
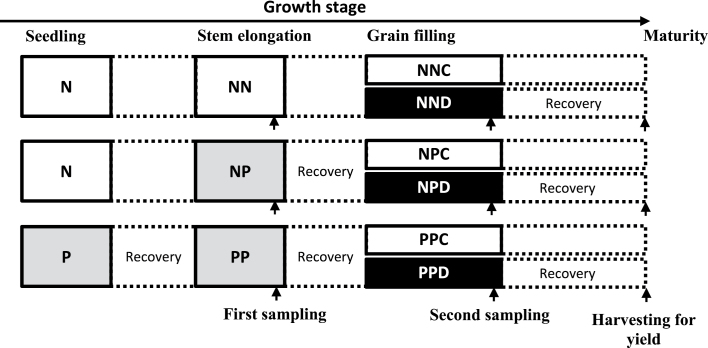
Diagram of the experimental design. Drought priming was applied either once during the stem elongation stage or twice during seedling and stem elongation. Drought priming was induced by withholding watering for 5–7 d until the soil water content reached approx. 35–40% for 2 d. Drought stress was applied 15 d after anthesis by withholding watering for 5 d until the soil water content dropped to 20–25% for 3 d. N, non-priming at the respective growth stage; P, priming at respective stage; NN, non-primed plants; NP, drought priming only at stem elongation stage; PP, drought priming at both seedling and stem elongation stages; NNC, control; NND, no priming+drought stress during grain filling; NPC, priming at stem elongation stage+non-stress during grain filling; NPD, priming at stem elongation stage+drought stress during grain filling; PPC, priming twice+non-stress during grain filling; PPD, priming twice+drought stress during grain filling. The small up arrows indicate time of sampling/measurement or harvesting. The primed plants and the drought-stressed plants during grain filling were rewatered immediately after the sampling and measurement.

The experimental design is shown in Fig. 1. Drought priming was applied either once, during the stem elongation stages (37 d after sowing, growth stage 39; see Lancashire *et al.*, 1991) or twice, during both seedling (27 d after sowing, growth stage 17) and stem elongation stages. The soil relative water content (SRWC) for the well-watered plants was set at 80–90%. Drought priming was applied by withholding watering for 5–7 d until the SRWC reached approx. 35–40% and maintained for 2 d. Drought stress was applied 15 d after anthesis by withholding watering for 5 d until the SRWC dropped to 20–25% and maintained for 3 d. The pots were weighed every day to monitor the soil water content at the target level. To avoid the heat stress and drought stress interaction, during drought priming and drought stress treatment, both the control plants and the drought plants were moved to a growth chamber (PGV36; Conviron, Montreal, Canada), at temperatures of 24/16 °C (day/night), a light intensity of 400 μmol photons m^–2^ s^–1^ and a humidity of 60%. After treatment, the pots were rewatered and moved outdoors until harvest maturity. There were no significant differences in phenological development of the plants among the treatments. Sampling and measurements were done at the end of drought priming at stem elongation and drought stress during grain filling. The last fully expanded leaves were used for physiological and proteome analysis during drought priming. Three treatments were included: non-primed plants (NN), drought priming applied at stem elongation stage (NP), and drought priming at both seedling and stem elongation stages (PP). The flag leaves were used for physiological and proteome analysis under drought stress during grain filling. Six treatments were included: control (NNC), no priming+drought stress during grain filling (NND), priming at stem elongation stage+non-stress during grain filling (NPC), priming at stem elongation stage+drought stress during grain filling (NPD), priming twice+non-stress during grain filling (PPC), and priming twice+drought stress during grain filling (PPD). All primed or drought-stressed plants were immediately rewatered after the sampling and measurements.

### SRWC and leaf relative water content (LRWC)

SRWC was calculated using the formula:

$$\begin{array}{l} \text{SRWC}\left(\%\right)=\left(\text{actual pot weight}–\text{pot weight with dried soil}\right)\\ \;\times \text{1}00/\left(\begin{array}{l} \text{pot weight with saturated soil}\\ –\text{pot weight with dried soil} \end{array}\right). \end{array}$$

LRWC was measured according to Jensen *et al.* (2000) and determined as follows:

$$\begin{array}{l} \text{LRWC}\left(\%\right)\text{=}(fresh\;weight\;–dried\;weight)\\ \;\times \text{1}00/(\text{fully turgid weight}–\text{dried weight}). \end{array}$$

Three biological replicates were analysed.

### Leaf gas exchange

Leaf gas exchange was measured as reported previously (Wang *et al.*, 2011) using an LI- 6400 portable photosynthesis system (LI-COR Biosciences, Lincoln, NE, USA) from 9:00 a.m. to 11:30 a.m. A standard 2×3cm chamber and light-emitting diode light source were used to support a constant photosynthetically active radiation level of 1000mol m^–2^ s^–1^. All measurements were taken at a constant flow rate of 500ml min^–1^ and a CO_2_ concentration of 400 μmol mol^–1^. Three biological replicates were analysed.

### Determination of malondialdehyde (MDA) content and ascorbate peroxidase (APX) activity

The extract for determination of the membrane lipid peroxidation was prepared according to Tan *et al.* (2008) and Jiang and Zhang (2002). Leaf sample (0.1g) was homogenized with a cold mortar and pestle in 3ml of extraction solution [50mM PBS (pH 7.0), 0.4% (w/v) polyvinylpyrrolidone] and with the addition of 1mM ascorbic acid for the APX assay. The homogenate was centrifuged at 12 000*g* for 20min at 4 °C, and the supernatant was collected and for further analysis.

A mixture of 1ml of supernatant and 4ml of reaction solution (thiobarbituric acid reactive substances (with 0.5% in 20% trichloroacetic acid)] were heated by incubating at 95 °C for 25min and immediately cooled in ice bath. The mixture was centrifuged at 12 000 *g* for 10min, and supernatant was used to determined MDA content at 532nm and 600nm. APX (EC 1.11.1.11) was assayed according to Nakano and Asada (1981). In brief, 1ml of reaction solution contained 50mM PBS (pH 7.0), 0.5mM ascorbic acid, 0.1mM H_2_O_2_, and 100 μl of extraction supernatant. APX activity was observed by recording the decreased rate of ascorbic acid oxidized at 290nm for 1min. Absorbance was measured with a UV/visible spectrophotometer (UltrospecTM 2100 *pro*; Amersham Biosciences). Three biological replicates were analysed.

### Protein extraction and quantification

Leaf protein extraction was performed according to Rinalducci *et al.* (2011), with minor modifications. Three biological replicates of fresh wheat leaves (0.5g) were finely ground in liquid nitrogen. The powder was suspended in 5ml of cold (–20 °C) 10% (w/v) trichloroacetic acid in acetone containing 0.07% (w/v) dithiothreitol (DTT) and one tablet per 50ml of extraction solution Protease Inhibitor Cocktail tablets (Roche), vortexed, and incubated at –20 °C overnight. The extraction was centrifuged at 35 000*g* for 1h at 4 °C. The collected pellet was washed with 5ml of chilled (–20 °C) acetone containing 0.07% (w/v) DTT, precipitated for 2h at –20 °C, and then centrifuged at 20 000*g* at 4 °C for 30min; the procedure was repeated three times to make sure the pellet was colourless. The supernatant was removed and the pellet was dried at 4 °C. The pellet was solubilized in a freshly prepared buffer containing 9M urea, 4% (w/v) CHAPS, 1% (w/v) DTT, and 1% (v/v) pH 4–7 ampholytes (GE Healthcare, Freiburg, Germany), 35mM Tris (Sigma) via incubation at 30 °C overnight with continuous stirring (Thermo mixer). The mixture was centrifuged at 12 000*g* at room temperature for 20min and the supernatant was analysed by two-dimensional gel electrophoresis. A small aliquot was used to determine protein concentration by a modified Bradford assay (Ramagli, 1999). Three biological replicates were analysed.

### Two-dimensional gel electrophoresis

Isoelectric focusing was performed with the Ettan^TM^ IPGphor (GE Healthcare) using IPG strips (linear pH 4–7, 18cm; GE Healthcare). Protein samples of 400 μg were added to a total volume of 350 μl of same solubilization solution containing 1% ampholyte pH 4–7 (GE Healthcare) and a trace of orange G. The solution was thoroughly vortexed and loaded on the strip. Isoelectric focusing was performed at 20 °C at a total of 67 kVh. IPG strips were subsequently equilibrated in 5ml of equilibration buffer [50mM Tris/HCl (pH 8.8), 6M urea, 30% (v/v) glycerol, 2% (w/v) SDS, 0.01% (w/v) bromophenol blue] with 1% (w/v) DTT (15min), followed by 5ml of equilibration buffer with 2.5% (w/v) iodoacetamide (15min). Separation in the second dimension was performed in 12.5% acrylamide (40% T, 3% C) gels using an Ettan^TM^ Daltsix Electrophoresis Unit (GE Healthcare) according to the manufacturer’s protocol. Strips together with a molecular marker (Mark 12^TM^; Invitrogen, Denmark) were placed on the gels and overlaid with 0.5% molten agarose. Separation was performed at 2W per gel (45min), followed by 12W per gel (4h) (until the dye front reached the gel bottom). Gels were stained overnight by colloidal Coomassie Brilliant Blue G-250 (Candiano *et al.*, 2004). Three biological replicates were analysed.

### Image analysis

Two-dimensional gels were scanned using a ScanMaker 9800XL (Microtek) at 300 dpi resolution in both colour and greyscale (16 bits). Spot detection and gel comparison were analysed using Progenesis SameSpots v.4.1 software (Nonlinear Dynamics, UK). The gel image from the control was chosen as a reference template, and spots in other gels were automated to match the reference gel and then edited manually to correct mismatched and unmatched spots. From the software, the average normalized spot quantity value was determined. The threshold of analysis of variance (ANOVA) was carried out at *P*≤0.05, power ≥0.8 and 1.5-fold change in average spot volume between treatments and the corresponding control was used to select the different spots for further mass spectrometry (MS) analysis. Spots had to be present on three replicate gels to be considered as present in a reproducible way.

### In-gel digestion and protein identification

Matrix-assisted laser desorption/ionization time-of-flight (MALDI-TOF) MS and MS/MS are commonly used for the protein identification of spots separated by two-dimensional electrophoresis gels (Jensen *et al.*, 1997; Rinalducci *et al.*, 2011; Huang *et al.*, 2012; Kausar *et al.*, 2013). The spots analysed by Progenesis SameSpots software were excised manually and subjected to in-gel trypsin digestion according to Yang *et al.* (2010). Aliquots (1 μl) of trypsin spot digests were applied to an Anchor Chip^TM^ target plate (Bruker-Daltonics, Bremen, Germany), covered by 1 μl of matrix solution (0.5g l^–1^ of α-cyano-4-hydroxycinnamic acid in 70% acetonitrile, 0.1% trifluoroacetic acid) and washed in 1 μl of 0.5% trifluoroacetic acid. Spectra were calibrated externally and internally using a trypic digest of β-lactoglobulin (5 pmol μl^–1^) and porcine trypsin autolysis products, respectively. The trypsin autolysis products (*m*/*z* 842.51 and 2211.10, respectively) were used as internal spectra calibration. Peptide mass fingerprinting and MS/MS data were acquired with Flex analysis 3.0 software (Bruker- Daltonics). Protein identification was performed by searching the NCBInr (National Center for Biotechnology Information, http://www.ncbi.nlm.nih.gov/) database using an in-house Mascot server (http://www.matrixscience.com) integrated with BioTools v3.1 software (Bruker-Daltonics). The following parameters were applied: taxonomic category: Green plant, allowed global modification, carbamidomethyl cysteine; variable modification, oxidation of methionine; missed cleavages, 1; peptide tolerance, 80 ppm; and MS/MS tolerance ±0.5Da. To be considered as a positive identification, the score of results had to be over the significance threshold level (*P*<0.05), and at least five matched independent peptides for peptide mass mapping were required. Sequences encoding proteins of unknown function were subjected to a BLAST (Basic Local Alignment Search Tool; http://www.ncbi.nlm.nih.gov/BLAST/) search in NCBI (Altschul *et al.*, 1990). Functional classifications of identified proteins were based on Bevan *et al.* (1998).

### Statistics

One-way ANOVA was applied to analyse the difference between treatments. Significant differences at *P*<0.05 among all treatments was determined by Duncan’s multiple range test (Sigmaplot 11.0; Systat Software).

## Results

### SRWC and LRWC

**Fig. 2.**
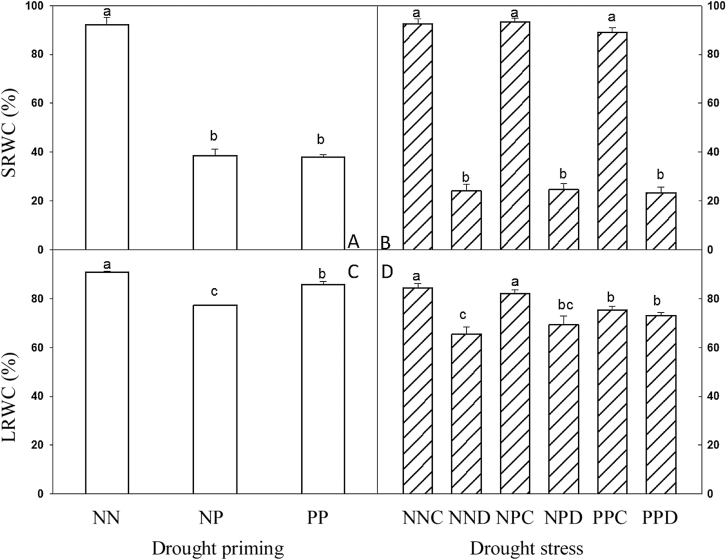
SRWC and LRWC under drought priming and drought stress. (A, B) SRWC under drought priming (A) and drought stress (B). (C, D) LRWC under drought priming (C) and under drought stress (D). See Fig. 1 legend for abbreviations. Different letters indicate significant differences at *P*<0.05 among all treatments as determined by Duncan’s multiple range test.

SRWC was maintained at around 37% during drought priming and at 25% under drought stress during grain filling (Fig. 2A, B). After drought priming, LRWC was lower in NP and PP compared with NN, and NP had a lower LRWC than PP (Fig. 2C). Under drought stress during grain filling, LRWC was lower in NND, NPD, PPC, and PPD, in relation to NNC (Fig. 2D).

### Grain yield and leaf photosynthesis

**Fig. 3.**
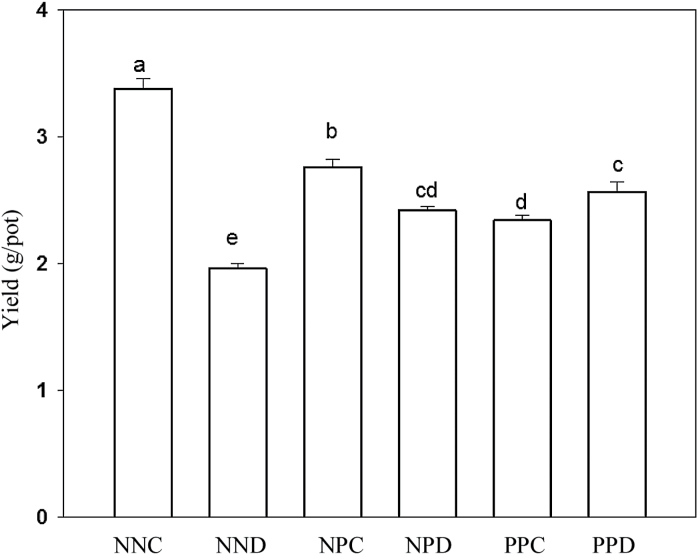
Effect of drought priming on grain yield in wheat experiencing drought stress after anthesis. See Fig. 1 legend for abbreviations. Three biological replicates were performed. Different letters indicated significant differences at *P*<0.05 among all treatments as determined by Duncan’s multiple range test.

Drought stress during grain filling (NND, NPD, and PPD) significantly decreased grain yield, in relation to NNC (Fig. 3). However, the primed plants (NPD and PPD) showed less yield reduction than non-primed plants (NND). In relation to NNC, grain yield was downregulated by 45, 30, and 31% in NND, NPD, and PPD, respectively. In addition, drought priming before anthesis treatments (NPC and PPC) significantly downregulated grain yield compared with NNC.

**Fig. 4.**
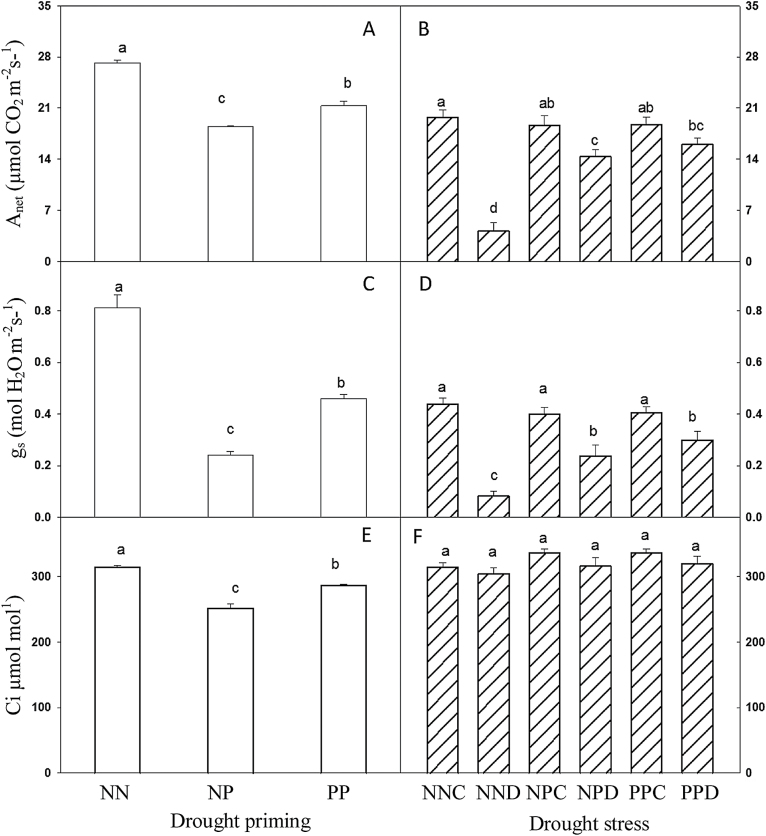
Effect of drought priming on net photosynthetic assimilation rate (*A*_net_), stomatal conductance (*g*_s_), and internal CO_2_ concentration (*C*_i_) of wheat leaves under drought priming and drought stress. (A, B) *A*_net_ under drought priming (A) and drought stress (B). (C, D) *g*_s_ under drought priming (C) and drought stress (D). (E, F) *C*_i_ under drought priming (E) and drought stress (F). See Fig. 1 legend for abbreviations. Three biological replicates were analysed. Different letters indicated significant differences at *P*<0.05 among all treatments as determined by Duncan’s multiple range test.

After drought priming, the photosynthetic assimilation rate (*A*_net_) was downregulated by 32 and 15% in NP and PP, respectively, in relation to NN (Fig. 4A). Under drought stress during grain filling, in relation to NNC, *A*_net_ decreased by 78, 26, and 17% in NND, NPD, and PPD, respectively. Thus, the drought-primed plants (NPD and PPD) showed significantly higher *A*_net_ than the non-primed plants (NND) under drought stress during grain filling (Fig. 4B). Stomatal conductance (*g*_s_) in NP and PP was downregulated by 70 and 47%, respectively, compared with NN (Fig. 4C). Drought stress also significantly reduced *g*_s_ in relation to NNC, while NPD and PPD showed significantly higher *g*_s_ than NND (Fig. 4D). Internal CO_2_ concentration (*C*_i_) was downregulated in NP and PP in relation to NN (Fig. 4E), while no significant differences were found under drought stress (Fig. 4F). There were no differences of *A*_net_ and *g*_s_ among NNC, NPC, and PPC.

### Leaf MDA content and APX activities

**Fig. 5.**
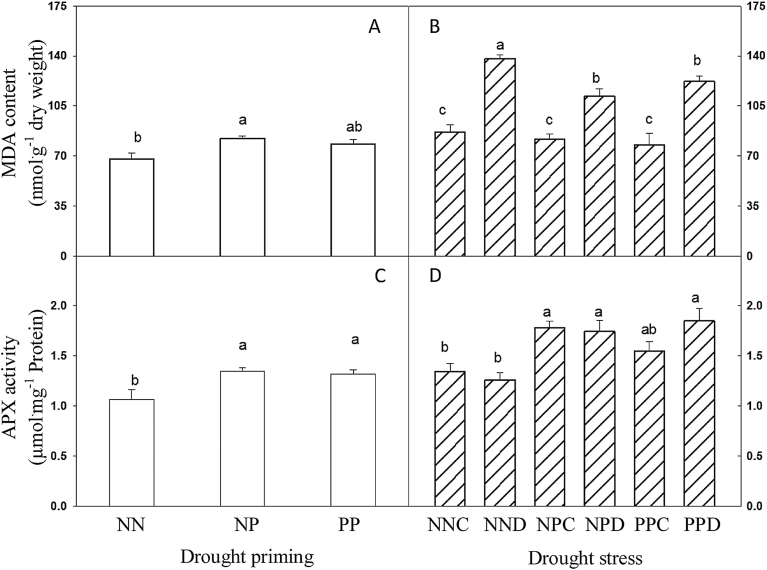
Effect of pre-anthesis drought priming on MDA content and APX activity in wheat leaves under drought priming and drought stress. (A, B) MDA content under drought priming (A) and drought stress (B). (C, D) APX activity under drought priming (C) and drought stress (D). See Fig. 1 legend for abbreviations. Three biological replicates were performed. Different letters indicated significant differences at *P*<0.05 among all treatments as determined by Duncan’s multiple range test.

Leaf MDA content was upregulated by 33 and 30% in NP and PP, respectively, in relation to NN. Drought stress upregulated the MDA content significantly, while the MDA content in NND, NPD, and PPD was upregulated by 59, 28, and 42% respectively, relative to NNC. There were no significantly differences of MDA content among NNC, NPC, and PPC (Fig. 5A, B).

After drought priming, APX activities were upregulated by 23 and 19% in NP and PP compared with NN. Under drought stress during grain filling, compared with NNC, no significant difference of the APX activity in NND was observed, while APX activity was upregulated by 30 and 38% in NPD and PPD, respectively (Fig. 5C, D). There were no significant differences in NPD and PPD compared with the corresponding control NPC and PPC. NPC and PPC showed higher APX activity in relation to NNC.

### Proteome profiles

**Fig. 6.**
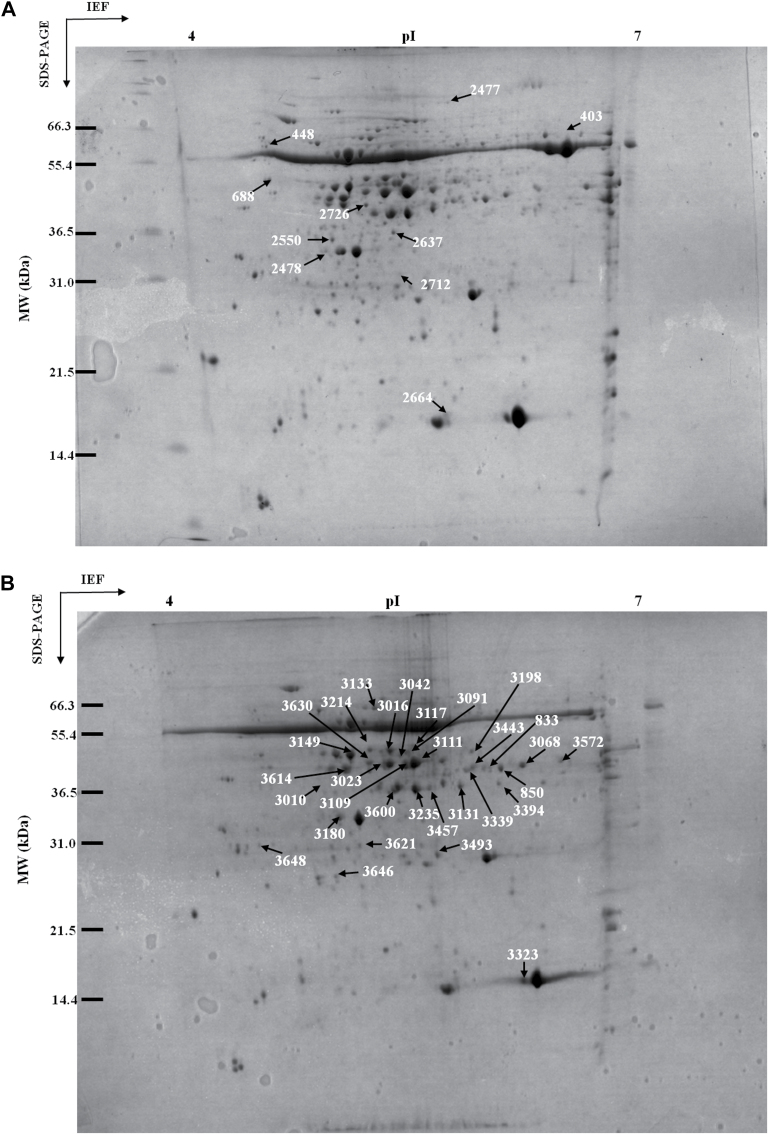
Representative two-dimensional electrophoresis gels of wheat leaf proteins during drought priming and drought stress. (A) Representative gel resulting from Progenesis Samespot software comparing the non-primed plants, stem elongation stage-primed plants, and twice-primed plants. (B) Representative gel resulting from Progenesis Samespot software comparing control plants, non-primed plants under drought stress, stem elongation-primed plants under drought stress, and twice-primed plants under drought stress. Differentially expressed protein spots in the drought priming treatments and drought stress treatments are indicated by arrows and listed in Tables 1 and 2. *M*_r_, relative molecular mass; pI, isoelectric point.

**Fig. 7.**
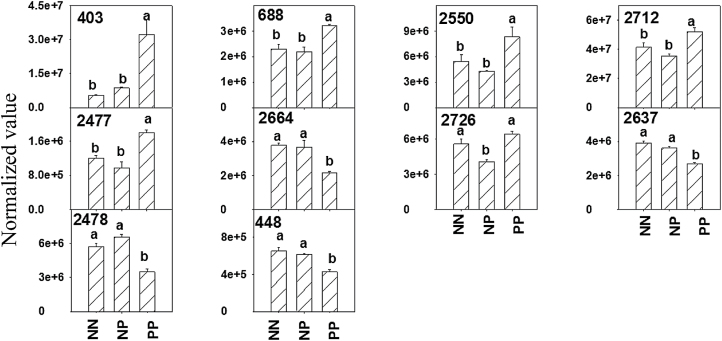
Quantitative variations (expressed as average normalized volumes) of spots differently expressed by drought priming. See Fig. 1 legend for abbreviations. Numbers are the same as spot numbers shown in Fig. 6A. Different letters indicate significant differences at *P*<0.05 among all treatments as determined by Duncan’s multiple range test.

Representative gels of protein expression profiles in wheat leaves after priming before anthesis and drought stress during grain filling are shown in Fig. 6. In total, approximately 600 and 400 protein spots were detected in samples after priming and drought stress, respectively, which corresponds well with previous studies (Budak *et al.*, 2013; Donnelly *et al.*, 2005; Fulda *et al.*, 2011). Ten protein spots were detected as differentially expressed between the drought priming treatments (NP and PP) and the non-priming treatment (NN) (Fig. 6A), of which seven were successfully identified (Table 1). These identified proteins were classified as being related to photosynthesis, stress defence, cell structure, and unknown function. Compared with NP, six spots (403, 688, 2550, 2712, 2477, and 2726) were upregulated and four spots (2664, 2637, 2478, and 448) were downregulated in PP (Fig. 7). The upregulated proteins included: Rubisco large subunit (RLS, spot 403), peptidyl-prolyl *cis*-*trans* isomerase CYP38 (spot 688), 4-nitrophenyl phosphatase (spot 2550), and PSII stability/assembly factor HCF136 (spot 2726). The downregulated proteins included the Rubisco small subunit (RSS, spot 2664), thioredoxin-like protein CDSP32 (spot 2637), and the putative myosin heavy chain (spot 2478).

**Table 1.** List of proteins differently expressed under drought priming analysed by MALDI-MS and MS/MS

Spot no.	FC^*a*^	Protein name	Accession no.	Taxonomy	Theor. *M*_r_^*b*^/pI	Exp. *M*_r_^*c*^/ pI	Match no.^*d*^	SC^*e*^	E-value	Peptides sequence	Function
Upregulated under drought priming											
403	6.1	Rubisco large chain	gi|14017580	*Triticum aestivum*	53.4/6.2	63/6.6	39	63	1.3E–09		Photosynthesis
688	1.5	Predicted: peptidyl-prolyl *cis*- *trans* isomerase CYP38	gi|357147646	*Brachypodium distachyon*	46.5/4.8	46/4.7	13	30	6.5E–07		Photosynthesis
2550	1.5	4-*nitrophenylphosphatase*	gi|226491816	*Zea mays*	39.5/5.5	35/5.1	13	26	4.1E–05		Unknown
2712	1.8	No match									
2477	1.5	No match									
Downregulated under drought priming											
2664	1.8	Rubisco small chain PW9	gi|132099	*Triticum aestivum*	19.8/8.5	17/5.8			7.0E–03	EYPDAYVR	Photosynthesis
									1.7E–05	EHNSSPGYYDGR	
2726	1.5	Photosystem II stability/ assembly factor HCF136	gi|357117071	*Brachypodium distachyon*	37.0/5.4	43/5.3	10	30	3.2E–03		Photosynthesis
2637	1.5	Thioredoxin-like protein CDSP32	gi|15222954	*Arabidopsis thaliana*	34.0/8.7	36/5.5			3.2E–04	GELIGEILR	Stress defence
2478	1.9	Putative myosin heavy chain	gi|20197623	*Arabidopsis thaliana*	86.2/4.9	34/5.1	14	15	1.5E–02		Cell structure
448	1.5	No match									

^*a*^The value represents the highest fold change of stem elongation-primed plants (NP) and twice-primed plants (PP) compared with non-primed plants (NN).

^*b*^Theoretical *M*_r_/pI (kDa).

^*c*^Experimental *M*_r_/pI (kDa) values were calculated using the Progenesis Samespot software.

^*d*^Match no. represents the number of identified peptides that can be matched with protein peptides in the database.

^*e*^Sequence coverage (%).

**Table 2.** List of proteins differently expressed under drought stress analysed by MALDI-MS and MS/MS

Spot no.	FC^*a*^	Protein name	Accession no.	Taxonomy	Theor. *M*_r_^*b*^/pI	Exp. *M*r^*c*^/pI	Match no.^*d*^	SC^*e*^	E-value	Peptides sequence	Function
Upregulated under drought stress											
3180	1.8	Oxygen-evolving enhancer protein 1	gi|357111487	*Brachypodium distachyon*	34.8/5.7	35/5.2	10	39	3.3E–07		Photosynthesis
3621	1.6	Ascorbate peroxidase	gi|226897533	*Triticum aestivum*	26.8/5.5	32/5.3	11	38	5.2E–05		Stress defence
3646	1.6	2-Cys peroxiredoxin BAS1	gi|2499477	*Hordeum vulgare*	23.4/5.5	28/5.2	6	32	5.1E–04		Stress defence
3010	2.7	Plastid glutamine synthetase isoform GS2c	gi|71362640	*Triticum aestivum*	47/5.8	43/5.1	6	17	8.4E–03		Protein synthesis
Downregulated under drought stress											
3600	1.5	Fructose-bisphosphate aldolase 2	gi|326499908	*Hordeum vulgare*	41.8/6.4	39/5.6	18	38	5.2E–08		Metabolism
3572	2.3	Fructose-bisphosphate aldolase, cytoplasmic isozyme 1-like	gi|326493652	*Hordeum vulgare*	38.1/6.1	42/6.7	9	21	2.1E–08		Metabolism
833	2.2	Fructose-bisphosphate aldolase, cytoplasmic isozyme 1-like	gi|326523629	*Hordeum vulgare*	41.6/7.1	42/6.2	7	11	1.4E–02		Metabolism
3023	2.5	Chloroplast fructose-bisphosphate aldolase	gi|223018643	*Triticum aestivum*	42.2/5.9	46/5.6	17	50	5.2E–12		Metabolism
3235	1.3	Fructose-bisphosphate aldolase, cytoplasmic isozyme 1-like	gi|326499908	*Hordeum vulgare*	41.7/6.4	37/5.7	17	38	3.7E–02		Metabolism
3493	1.7	Triosephosphate-isomerase	gi|326496613	*Hordeum vulgare*	32.7/7.0	30/5.8	9	28	6.4E–03		Metabolism
850	1.9	Cytosolic malate dehydrogenase	gi|37928995	*Triticum aestivum*	24.6/6.6	41/6.3	6	23	1.3E–02		Metabolism
3042	1.5	Phosphoglycerate kinase	gi|129915	*Triticum aestivum*	50/6.6	48/5.6			4.7E–05	SVGDLTAADLEGKR	Metabolism
3323	1.9	Rubisco small subunit	gi|11990901	*Triticum aestivum*	19.7/8.8	16/6.3	9	50	2.1E–05		Photosynthesis
3068	2.5	Rubisco activase small isform	gi|313574196	*Hordeum vulgare*	47.3/7.6	42/6.4	15	38	5.1E–10		Photosynthesis
3109	2.2	Rubisco activase B	gi|7960277	*Triticum aestivum*	48/6.9	45/5.7	12	34	2.9E–03		Photosynthesis
3198	1.8	Rubisco activase	gi|37783283	*Triticum aestivum*	22.5/5.0	47/6.1	11	51	1.3E–10		Photosynthesis
3111	2.4	Rubisco activase alpha form precursor	gi|32481061	*Deschampsia antarctica*	51.4/6.0	45/5.7	23	44	5.1E–11		Photosynthesis
3214	2.8	Rubisco activase alpha form precursor	gi|32481061	*Deschampsia antarctica*	51.4/6.0	50/5.4	16	37	1.6E–09		Photosynthesis
3016	2.5	Rubisco activase alpha form precursor	gi|32481061	*Deschampsia antarctica*	51.4/6.0	50/5.6	12	21	4.1E–07		Photosynthesis
3443	1.7	Rubisco activase	gi|37783283	*Triticum aestivum*	22.5/5.0	42/6.1	7	24	9.9E–04		Photosynthesis
3091	1.5	Rubisco activase small isoform	gi|313574196	*Hordeum vulgare*	47.3/7.6	49/5.7	16	42	2.9E–03		Photosynthesis
3117	1.6	Actin	gi|168472715	*Lolium temulentum*	41.2/5.5	51/5.7	12	31	2.1E–10		Cell structure
3133	2.3	Rubisco large subunit-binding protein subunit beta	gi|2493650	*Secale cereale*	53.7/4.9	61/5.5	12	26	6.6E–08		Molecular chaperones
3149	1.6	Plastid glutamine synthetase isoform GS2c	gi|71362640	*Triticum aestivum*	47/5.8	49/5.3	19	47	3.3E–11		Protein synthesis
3394	1.9	Cysteine synthase	gi|585032	*Triticum aestivum*	34.2/5.5	36/6.3	11	32	5.2E–05		Protein synthesis
3131	2.6	Cysteine synthase	gi|585032	*Triticum aestivum*	34.2/5.5	37/6.1	11	31	2.6E–06		Protein synthesis
3457	1.8	Cysteine synthase	gi|585032	*Triticum aestivum*	34.2/5.5	37/5.8	13	46	1.6E–06		Protein synthesis
3648	1.7	29kDa ribonucleo protein	gi|326510421	*Hordeum vulgare*	29.6/4.8	33/4.6			1.6E–02	VNSGPPPPRDEFAPR	Unknown
3339	1.6	Hypothetical protein MTR_026s0001	gi|358343995	*Medicago truncatula*	12.7/10	41/6.1	6	54	2.1E–03		Unknown

^*a*^The value represents the highest fold change of non-primed plants under drought stress (NND), stem elongation-primed plants under drought stress (NPD) and twice-primed plants under drought stress (PPD) compared with control (NNC).

^*b*^Theoretical *Mr/pI* (kDa).

^*c*^Experimental *Mr/pI* (kDa) values were calculated using the Progenesis Samespot software.

^*d*^Match no. represents the number of identified peptides that can be matched with protein peptides in the database.

^*e*^Sequence coverage (%).

**Fig. 8.**
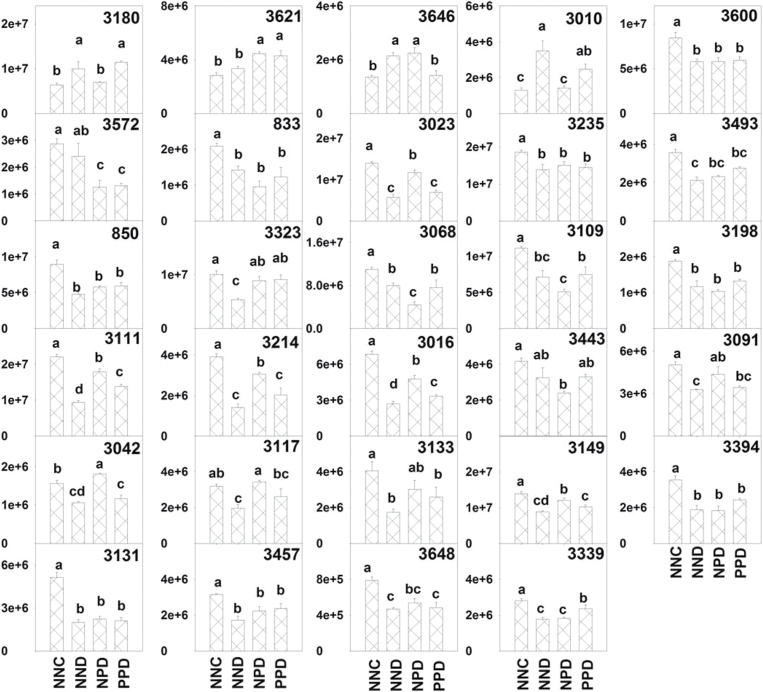
Quantitative variations (expressed as average normalized volume) of spots identified as differently expressed between primed plants and non-primed plants under drought stress. See Fig. 1 legend for abbreviations. Numbers are the same as spot numbers shown in Fig. 6B. Different letters indicate significant differences at *P*<0.05 among all treatments as determined by Duncan’s multiple range test.

**Fig. 9.**
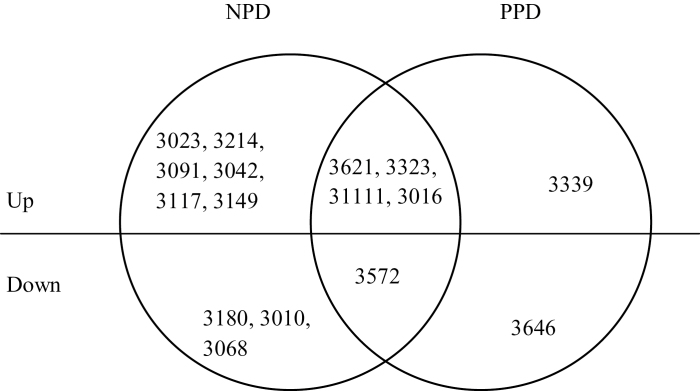
Venn diagrams of changed protein spots in primed plants (NPD and PPD) compared with non-primed plants (NND) under drought stress during grain filling. See Fig. 1 legend for abbreviations. The numbers indicate differently expressed protein spots.

Under drought stress during grain filling (NND, NPD, and PPD), 29 proteins were differently expressed and successfully identified as compared with NNC (Fig. 6B), and of these, 25 proteins spots were downregulated and four were upregulated by drought stress. The differential expression abundances are shown in Fig. 8. The identified proteins were classified as photosynthesis, stress defence, metabolism, molecular chaperone, protein synthesis, cell structure, and unknown function (Table 2). The protein spots identified as Rubisco activase (RCA, spots 3111 and 3016), RSS (spot 3323), and APX (spot 3621) were commonly upregulated, while the spot identified as fructose-bisphosphate aldolase (FBA, spot 3572) was downregulated in primed plants compared with the non-primed plants (Fig. 9). In relation to NPD, spots identified as phosphoglycerate kinase (spot 3042), actin (spot 3117), glutamine synthetase (spot 3149), and 2-Cys peroxiredoxin BAS1 (spot 3646) were downregulated in PPD, while spots identified as glutamine synthetase (spot 3010), oxygen-evolving enhancer proteins (spot 3180), and Hypothetical protein MTR_026s0001 (spot 3339) were upregulated in PPD (Fig. 9). Two spots (3010, 3149) identified as glutamine synthetase showed contrasting expression profiles under drought stress during grain filling. Spot 3149 was upregulated in NPD compared with NND and PPD. Spots 3010 and 3149 had similar pI values (5.1 and 5.3), while the lower molecular weight of spot 3010 (43kDa) compared with spot 3149 (49kDa) might suggest that 3010 is a fragment or another form of glutamine synthetase (spot 3149).

## Discussion

It is known that epigenetic modifications are involved in *trans*-generational stress memory (Molinier *et al.*, 2006; Schmitz *et al.*, 2011). However, it is not clear whether the mechanisms of epigenetic modifications (histone and DNA modifications) are involved in stress memory within the same generation (Chinnusamy and Zhu, 2009). It has been shown that accumulation of mRNA and proteins of MPKs are involved in signalling mechanisms for pathogen priming. In particular, the upregulation of activities of MPK3 and MPK6 were found to enhance the immune response in *Arabidopsis* (Beckers *et al.*, 2009; Conrath, 2011). However, the mechanisms of abiotic stress-based priming on subsequent abiotic stress events are far from clear. The objective of this study was to study the effect and potential mechanisms of drought priming before anthesis on drought stress episodes occurring during grain filling. Both physiological and proteome studies were performed to elucidate differences between primed and non-primed plants under drought stress during grain filling, which may contribute to improved drought tolerance.

### Response of priming on physiological traits in leaves and on grain yield

LRWC is often used as an index of the water status of the plant (Sinclair and Ludlow, 1986; Ali *et al.*, 1999), and can be used as an indicator for drought tolerance in wheat (Loutfy *et al.*, 2012). In this study, primed plants showed higher stomatal conductance and higher leaf water content than non-primed plants. Stomatal control is important for regulation of both water loss and CO_2_ assimilation in response to drought stress (Jones, 1998; Munns *et al.*, 2010). Here, the higher stomatal conductance in primed than in non-primed plants was in accordance with the higher rates of photosynthesis. It has been shown that abscisic acid (ABA)-based chemical signals may regulate physiological responses to drought stress (Socias *et al.*, 1997; Dodd, 2005), and that increased leaf ABA concentrations would lead to lower stomatal conductance. In this study, under drought priming, ABA and *g*_s_ showed a negative correlation (Supplementary Fig. S1B at *JXB* online). However, there was no correlation between ABA and *g*_s_ under drought stress during grain filling (Supplementary Fig. S2B at *JXB* online). Thus, the higher ABA concentration under grain filling might not have been responsible for stomatal closure. Instead, the higher leaf water status could have allowed the stomata to remain open in primed plants. In line with this, in drought-stressed soybean, Liu *et al.* (2004) showed that exogenous ABA application during drought could enhance stomatal conductance and leaf water potential as compared with non-ABA-treated plants.

As alternative functions of ABA in abiotic stress response, ABA can induce genes encoding dehydration proteins (Zhu, 2002) as well as the transcription of heat-shock proteins (Larkindale and Huang, 2004). In addition, it has been shown that accumulation of ABA can also trigger the generation of ROS leading to upregulation of activities of antioxidant enzymes (Jiang and Zhang, 2002; Penfield, 2008).

This was in accordance with our results showing a positive correlation of ABA with activities of APX under drought stress during grain filling (Supplementary Fig. 2A). Primed plants showed higher leaf ABA content, higher APX activity, and higher grain yield compared with non-primed plants. In other experiments, ABA levels in flag leaves during grain filling were higher in tolerant than in sensitive varieties, and have led to higher grain yield through regulation of assimilate partitioning into grains (Guóth *et al.*, 2009).

In this study, the LRWC of primed plants PPD was significantly higher than in non-primed plants (NND), and was higher in primed plants (PPD and NPD). The better maintenance of leaf water status in primed plants than in non-primed plants may have contributed to the better photosynthetic performance. Thus, the better maintenance of leaf water status could be a consequence of the priming effect, contributing to improved drought tolerance under drought stress during grain filling in primed compared with non-primed plants. Furthermore, biochemical mechanisms also contribute to drought tolerance under prolonged drought stress (Costa França *et al*., 2000).

Photosynthesis is the primary process affected by water deficit and can lead to reductions in crop yield (Chaves, 1991; Flexas *et al.*, 2004; Chaves *et al.*, 2008). Our previous studies found that heat priming alleviated the inhibition of photosynthesis under a subsequent heat-stress episode (Wang *et al.*, 2011, 2014). In this study, there were no significant differences in the photosynthesis rate between primed plants under non-stress (NPC and PPC) and non-primed plants under non-stress during grain filling (NNC). However, primed plants under drought stress (NPD and PPD) showed significantly higher photosynthesis rates than did non-primed plants under drought stress (NND), suggesting that primed plants showed a higher capacity to protect photosynthetic activity in response to a later drought stress rather than the establishment of an altered state. It is known that mild drought stress lowered photosynthetic rate by stomatal limitations, while both stomatal and non-stomatal limitations occur during severe drought stress (Flexas *et al.*, 2004; Lawlor and Tezara, 2009; Sengupta *et al.*, 2011). Non-stomatal limitation is attributed to reduced carboxylation efficiency (Feller *et al.*, 1998), reduced ribulose-1,5-bisphosphate regeneration (Kubien and Sage, 2008) or inhibition of RCA (Bayramov *et al.*, 2010). According to Farquhar and Sharkey (1982), a decrease in *g*_s_ and *C*_i_ means that stomatal limitation is responsible for the decrease in photosynthesis, while a decrease in both photosynthesis rates and *g*_s_ but higher *C*_i_ would indicate non-stomatal limitation inhibiting the CO_2_ assimilation. In accordance with this, in our experiment, it seems that the stomatal limitation occurred during drought priming, while under drought stress during grain filling the decrease in both the photosynthesis rate and *g*_s_ with no difference in *C*_i_ during drought stress may indicate the non-stomatal limitation of photosynthesis. The higher photosynthesis rates in primed plants under drought stress during grain filling (NPD and PPD) may indicate less limitation of both stomatal and non-stomatal factors. Inhibition of photosynthesis activity usually leads to higher production of ROS, which may cause oxidative damage to the photosynthetic apparatus, proteins, DNA, and lipids (Xiong *et al.*, 2002). MDA content is often used as an indicator of the extent of lipid peroxidation by ROS (Sairam *et al.*, 2000; Wang *et al.*, 2014). We have reported that heat priming through upregulation of both the activities and gene expression of superoxide dismutase, glutathione reductase, and peroxidase could alleviate oxidative damage under subsequent heat stress in wheat (Wang *et al.*, 2011, 2014). To counteract ROS damages, APX plays an important role by catalysing the conversion of H_2_O_2_ to H_2_O (Mittler, 2002). It has been reported that drought-acclimated plants showed a higher APX activity, which enhanced oxidative stress tolerance compared with non-acclimated plants (Selote and Khanna-Chopra, 2006). In accordance with this, our results demonstrated that, with drought stress during grain filling, primed plants showed higher APX activity in relation to non-primed plants, which is in accordance with lower MDA content. These results indicated that priming induced upregulation of APX activity to alleviate oxidative damage under drought stress during grain filling.

Drought stress is one of the limiting factors for the wheat yield production (Li P *et al*., 2011), especially when it happened during the reproductive growth stage (Barnabas *et al.*, 2008). However, it has been observed that plants that experienced drought stress during vegetative stage showed higher grain yield than non-acclimated plants under drought stress during the flowering stage (Yang *et al.*, 2011; Zhang *et al.*, 2013). In this study, grain yield was significantly downregulated by drought stress, while the higher grain yield in primed plants may have resulted from the higher rates of photosynthesis and lower oxidative damage during grain filling compared with the non-primed plants. The reduction in grain yield (and no difference in rates of photosynthesis) under non-stress during grain filling (NPC and PPC) may be because pre-anthesis drought priming decreased kernel numbers (unpublished data). The higher yield in PPD than PPC might be related to drought stress-induced senescence that enhanced the remobilization of pre-stored assimilates from vegetative organs to gains (Plaut *et al.*, 2004).

### Response of priming on the leaf proteome

#### Drought priming

RSS and RLS are both important components of Rubisco, the key enzyme involved in photosynthetic CO_2_ assimilation (Caruso *et al.*, 2009). There have been contrasting results in different studies on the regulation of Rubisco in response to drought stress, as some studies found upregulation (Zhou *et al.*, 2011; Budak *et al.*, 2013), some reported downregulation (Ali and Komatsu, 2006; Plomion *et al.*, 2006), and some reported both up- and downregulation (Caruso *et al.*, 2009). In this study, the protein abundances of RSS and RLS were differently expressed in PP compared with NN and NP. Since the photosynthesis rate in PP was higher than in NP, and the lower photosynthesis rate may result from stomatal limitation, it is suggested that the Rubisco activity may have not been significantly changed under mild drought stress during priming. Peptidyl-prolyl *cis*-*trans* isomerase CYP38, which is essential for PSII assembly (Fu *et al.*, 2007), and PSII stability/assembly factor HCF136, which is involved in assisting the folding of proteins and is required for the assembly and stabilization of PSII, were upregulated in PP but downregulated in NP in relation to NN. The upregulation of these proteins may indicate a protective role of the photosynthetic apparatus in PP compared with NP, which is in accordance with the higher photosynthesis rate in PP than in NP.

Thioredoxin-like protein CDSP32, which is a chloroplastic drought-induced stress protein of 32kDa, was induced by oxidative stress (Broin *et al.*, 2000). Here, after drought priming, Trx-CDSP32 was downregulated in PP in relation to NP. This may due to the absence of oxidative damage in PP under drought priming, as exemplified by no significant difference in MDA content between PP and NN. Putative myosin heavy chain has been identified in plants in response to heavy metal (Ahsan *et al.*, 2007) and cold stress (Yan *et al.*, 2006). However, the role of this protein is not clear. While the putative myosin heavy chain was downregulated in the PP treatment in this study, we were unable to relate the function of this protein to drought priming. As this protein was expressed in fully expanded leaves, it might be related to actin organization, organelle movement or signal transduction (Sparkes, 2011). Collectively, the different protein expression between PP and NP might be result of the priming effect during the seedling stage, which could be contributing to enhancing tolerance to mild drought stress during the stem elongation stage.

#### Drought stress during grain filling

The protein spots identified as RCA, RSS, and APX were upregulated, and FBA was downregulated in primed plants (NPD and PPD) compared with non-primed plants (NND), suggesting that these proteins may be involved in drought tolerance by priming. RCA removes inhibitors from the catalytic sites of Rubisco (Portis, 1995) and is reported to be impaired under water-deficit conditions (Tezara *et al.*, 1999). Under drought stress during grain filling, eight proteins spots were identified as RCA, and have also been found in other plant proteome studies (Ciais *et al.*, 2005; Donnelly *et al.*, 2005; Caruso *et al.*, 2009). These additional spots identified as one protein might be due to cleaved isoforms of the same protein (Hurkman *et al.*, 1994) or post-translational modified isoforms (Weiss and Görg, 2007). Here, two spots identified as RCA and one spot identified as RSS were significantly more highly expressed in primed plants (NPD and PPD) than in non-primed plants (NND). The higher RSS and RCA abundance in primed plants (NPD and PPD) indicated the contribution to higher photosynthesis rate compared with non-primed plants (NND) under drought stress during grain filling.

Changes in both amounts and activities of APX have been identified as an indicator of a redox status to counteract ROS damage under water deficit (Mittler, 2002). The upregulated expression of APX protein, higher activities of APX, and lower MDA content in primed plants compared with non-primed plants in the present study indicated a higher capacity for ROS scavenging and lower cell lipid peroxidation. This may indicate that primed plants can activate the antioxidant defence system when subjected to subsequent drought stress episodes.

It has been reported that increased photosynthesis and antioxidative defence-related proteins play important roles in hybrid Bermuda grass to adapt to drought stress (Zhao *et al.*, 2011). In our study, the differences in protein expression between primed plants (NPD and PPD) and non-primed plant (NND) indicated that photosynthesis and antioxidant defence-related proteins may play important roles in priming and drought stress during grain filling.

In our study, we did not find regulation of proteins related to signal transduction may be because the signalling proteins usually are only induced at the very early stage of stress sensing (maximum one day) and may not be related to long-lasting stress tolerance (Harb *et al.*, 2010; Pastor *et al.*, 2012).

FBA exist in two isoforms, a chloroplastic FBA and a cytosolic FBA, and are involved in gluconeogenesis and glycolysis (Schnarrenberger and Krüger, 1986; Michelis and Gepstein, 2000). In this study, two spots were identified as FBA, and one of them was downregulated in primed plants, while the other one was upregulated in NPD compared with PPD and non-primed plants, suggesting that FBA was regulated by drought stress. Several spots identified as FBA showing different expression have also been found in other studies (Xu and Huang, 2008; Zhao *et al.*, 2011). The upregulation of proteins involved in glycolysis, such as FBA and phosphoglycerate kinase (Houston *et al.*, 2009), in NPD compared with PPD indicated the higher energy demand in NPD under drought stress. In accordance, glutamine synthetase, which plays an important role in nitrogen metabolism (Caruso *et al.*, 2009), was expressed more highly in NPD than in PPD. This might indicate a higher ATP demand for nitrogen metabolism in NPD than in PPD.

The 2-Cys peroxiredoxin BAS1 is the target of the Trx-CDSP32, which plays roles in protecting the photosynthetic apparatus against oxidative damage (Broin *et al.*, 2002). Oxygen-evolving enhancer protein is a manganese-stabilizing protein for PSII core stability (Yi *et al.*, 2005). Actin, which is involved in the cell structure, was upregulated in NPD compared with PPD. The higher expression of proteins related to actin and 2-Cys peroxiredoxin BAS1 but lower expression of oxygen-evolving enhancer protein 1 in NPD compared with PPD may be related to the measured higher expression of PSII stability related proteins (peptidyl-prolyl *cis*-*trans* isomerase CYP38 and PSII stability/assembly factor HCF136) and cell structure-related protein (putative myosin heavy chain) but lower expression of Trx-CDSP in NP compared with PP under drought priming. These results suggest that these differences may have been ‘conserved’ from drought priming rather than induced by drought stress during grain filling.

## Conclusions

The single or double drought priming events before anthesis resulted in higher grain yield under drought stress during grain filling. The primed plants showed higher leaf water status, higher photosynthesis rates, higher APX activity, and lower cell membrane peroxidation than did the non-primed plants. Furthermore, the protein abundances of RSS, RCA, and APX were upregulated in primed plants compared with non-primed plants. Both the upregulated synthesis (expressed as protein abundance) and activities of proteins involved in photosynthesis and stress defence in primed plants could be contributing to the priming effects enabling the plants to cope with the drought stress during grain filling. In addition, proteins involved in general metabolism (glycolysis and nitrogen metabolism) were differently expressed in plants primed once or twice under drought stress, which might indicate that these processes are differently regulated.

## Supplementary Material

Supplementary Data

## Abbreviations:

ABA: abscisic acid
ANOVA: analysis of variance
*A*_net_: net photosynthetic assimilation rate
APX: ascorbate peroxidase
*C*_i_: internal CO_2_ concentration
DTT: dithiothreitol
FBA: fructose-bisphosphate aldolase
*g*_s_: stomatal conductance
LRWC: leaf relative water content
MALDI-TOF MS: matrix-assisted laser desorption/ionization time-of-flight mass spectrometry
MDA: malondialdehyde
MPK: mitogen-activated protein kinase
PS: photosystem
RCA: Rubisco activase
RLS: Rubisco large subunit
ROS: reactive oxygen species
RSS: Rubisco small subunit
Rubisco: ribulose-1,5-bisphosphate carboxylase/oxygenase
SRWC: soil relative water content.
