# Supplementary material for: Improved tolerance to drought stress after anthesis due to priming before anthesis in wheat (Triticum aestivum L.) var. Vinjett
Source: J Exp Bot. 2014 Sep 9;65(22):6441–56. doi: 10.1093/jxb/eru362 (PMC4246180; doi:10.1093/jxb/eru362)
Supplement: Supplementary Data [file supp_65_22_6441__index.html]

Improved tolerance to drought stress after anthesis due to priming before anthesis in wheat (Triticum aestivum L.) var. Vinjett — Improved tolerance to drought stress after anthesis due to priming before anthesis in wheat (Triticum aestivum L.) var. Vinjett — Supplementary Data 

# Improved tolerance to drought stress after anthesis due to priming before anthesis in wheat (*Triticum aestivum* L.) var. Vinjett

## Supplementary Data

Data files

**Files in this Data Supplement:**

- Supplementary Data - Supplementary Data
