# Supplementary material for: Improved tolerance to drought stress after anthesis due to priming before anthesis in wheat (Triticum aestivum L.) var. Vinjett
Source: J Exp Bot. 2014 Sep 9;65(22):6441–56. doi: 10.1093/jxb/eru362 (PMC4246180; doi:10.1093/jxb/eru362)
Supplement: Supplementary Data [file supp_eru362_jexbot108241_file001.pdf]

Supplementary Figures for ms JEXBOT/2013/108241

Title:

Improved tolerance to drought stress after anthesis due to priming before anthesis in wheat (*Triticum aestivum* L.) var. Vinjett

Authors:

Xiao Wang, Marija Vigjevic, Dong Jiang, Susanne Jacobsen, Bernd Wollenweber

Supplementary Figure Legend

Supplemental Fig.1 The correlation of ABA and APX (A), ABA and gs (B) under drought priming during the vegetative growth stages. r is the correlation coefficient. * indicate statistically significant differences ($P<0.05$).

Supplemental Fig.2 The correlation of ABA and APX (A), ABA and gs (B) under drought stress during grain filling. r is the correlation coefficient. * indicate statistically significant differences ($P<0.05$).

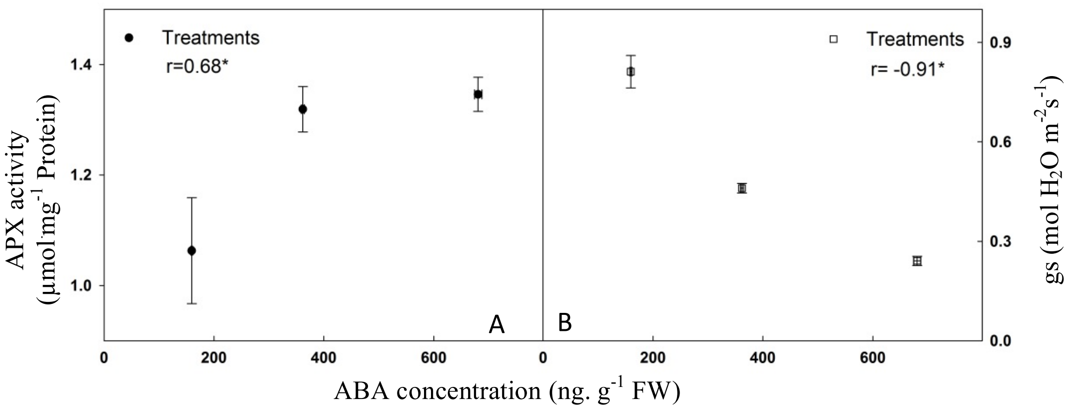

Supplementary Figure 1

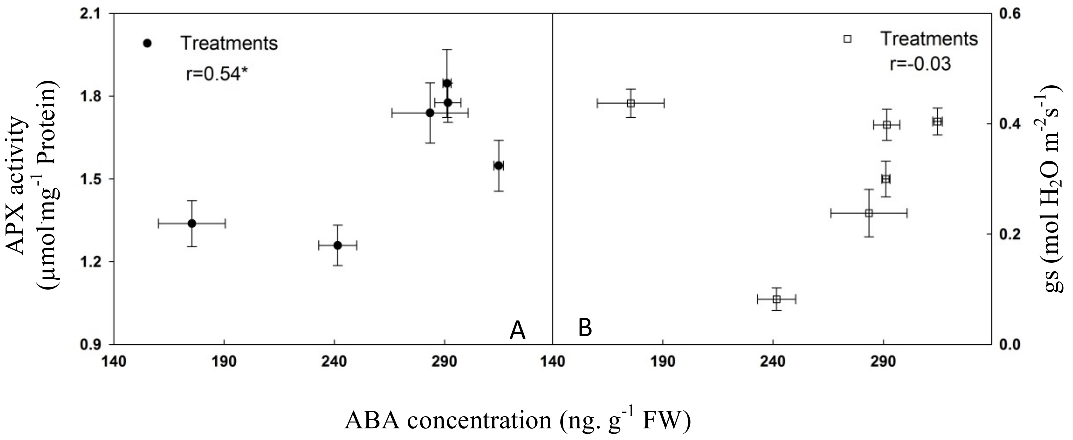

Supplementary Figure 2
